# Abalone Mortality Associated With Hypoxia in Tidepools During a Summer Heatwave

**DOI:** 10.1002/ece3.74126

**Published:** 2026-08-11

**Authors:** Marthe M. Gagnon, Graham L. Cobby, Fred E. Wells

**Affiliations:** ^1^ School of Molecular and Life Sciences Curtin University Bentley Western Australia Australia; ^2^ Negaunee Integrative Research Center Field Museum of Natural History Chicago Illinois USA

**Keywords:** Haliotidae, heatwave, marine invertebrates, mortality, Western Australia

## Abstract

Marine heatwaves are increasing in frequency, duration, and intensity, posing a growing threat to coastal ecosystems and contributing to widespread mortality of marine organisms. Along the intertidal rocky platforms of south‐western Western Australia, extreme low tides coinciding with heatwave conditions can expose habitats to elevated temperatures and prolonged periods of limited water exchange. The Roe's abalone ( *Haliotis roei* ), a species of commercial and recreational importance, occurs both on exposed platform surfaces and within intertidal tidepools. During a marine heatwave, substantial mortality of *H. roei* was observed within tidepools, whereas little to no mortality was recorded among individuals inhabiting adjacent platform surfaces. Measurements of seawater temperature indicated similar thermal conditions between tidepools and the platform edge. In contrast, dissolved oxygen concentrations were markedly reduced within tidepools, reaching severely hypoxic levels. These observations suggest that oxygen depletion, potentially acting in combination with heatwave conditions and restricted water exchange, may have contributed to the observed mortality of *H. roei* within tidepool habitats during the heatwave event.

## Graphical abstract

Abalone ( *Haliotis roei* ) inhabit tidepools on intertidal platforms. During heatwaves and periods of low tide, water exchange in tidepools ceases. While temperatures in tidepools remain constant, oxygen levels are significantly reduced. Abalone mortality in tidepools is associated with oxygen depletion rather than thermal stress.

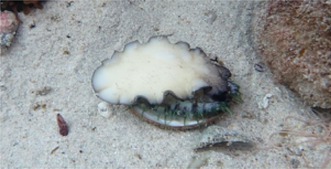

The coastline of Perth, Western Australia (WA) is essentially a long sandy beach interspersed with small, < 100 m wide, intertidal limestone platforms. The beach is depauperate because of the medium intensity wave action and constantly shifting sand (Ince et al. 2007; Morrison 2015). In contrast, the platforms are localised biodiversity hotspots, with > 130 species of molluscs recorded (Wells et al. 2023, 2024). The molluscs are predominately temperate species, except for the mussel *Brachidontes ustulatus* that forms dense mats on the platform at Trigg Point (Wells et al. 2024). The temperate abalone *Haliotis roei* is abundant at the seaward margins of the platforms and is a commercially and recreationally important species in the area (Hart et al. 2018; Strain et al. 2024).

The region is microtidal, a mixture of diurnal and semidiurnal tides; tidal ranges vary from a few centimetres to up to 1 m. Rock platforms occur from tidal datum to about 1 m above; many are at about 0.3 m. The platform surface is generally flat, but highly dissected by channels and pools on some platforms. There is usually a low ridge, or rim, up to 0.2 m high and 2 m wide on the seaward margin or along channels that trap water in shallow pools on the platform. As seawater drains from the platform at low tide it is replaced by waves crashing over the seaward rim or water emerging from fissures and blowholes in the rock surface (Wells and Walker 1993).

During winter, the prevailing south‐westerly winds and winter storms raise mean sea levels in the Perth area and the platforms are completely covered with seawater. In summer, wind patterns change to be predominantly easterly overnight and in the early morning. Mean sea level is 0.3 m lower in summer, and if a strong, hot easterly wind combines with a lunar low tide, the intertidal platforms may remain exposed for hours, contributing to mass mortalities of intertidal organisms, including abalone (Hodgkin 1959; Wells and Walker 1993; Kohn 1993; Wells et al. 2025).

Large intertidal platforms occur at Radar Reef and Cape Vlamingh at the west end of Rottnest Island, 20 km offshore from Perth. The Leeuwin Current flows southward along the outer continental shelf, providing a mechanism for tropical species to occur much further than would be expected (Pearce and Walker 1991; Pearce and Feng 2007). Radar Reef and Cape Vlamingh had a mixture of tropical, temperate, and WA endemic molluscs in 2007, including *H. roei* which was recorded in high abundance on the seaward margins of these platforms (Wells et al. 2024). In recent decades there have been a number of marine heatwaves on the west coast of WA. The most extensive was in the summer of 2010/11 (Pearce and Feng 2013) which caused considerable changes to the marine environment of the WA west coast (Wernberg et al. 2013, 2016; Caputi et al. 2019). A 2021 survey demonstrated that intertidal molluscs at Radar Reef and Cape Vlamingh had decreased by 90% and 86% respectively since a similar survey in 2007, with no *H. roei* recorded at these sites in 2021. This was thought to have been caused by the 2010/11 marine heatwave (Wells et al. 2023). The lack of recovery from heatwave events suggests that *H. roei* is sensitive to high temperatures when exposed on the intertidal platforms. The Leeuwin Current does not reach the continental shoreline in the Perth region and shoreline reefs were not affected by the 2010/2011 heatwave and remain highly biodiverse with abundant *H. roei* (Wells et al. 2024).

In February 2024, during mollusc mortality monitoring at Cottesloe, Trigg Point, and Waterman, the first case of abalone mortality in tidepools during a heatwave was observed and documented at Waterman. Individuals were considered to be moribund if they reacted slowly when probed or dead if there was no reaction.

During this heatwave that coincided with low morning tides, water physical parameters oxygen saturation (%) and temperature (°C) were measured at Waterman (−31.854490, 115.751378) at the seaward edge of the platform and at the bottom of three shallow intertidal pools (Figure 1). The three tidepools had the following dimensions (LxWxD): 180 × 70 × 35; 170 × 110 × 75; 130 × 70 × 34 cm. Observations were made in the same three tidepools over the heatwave period. Measurements were taken on 7, 8 and 9 February 2024 with an Oxyguard Polaris C dissolved oxygen meter. Salinity was measured with an Automatic Temperature Compensation handheld salinity refractometer. Within each of the three tidepools, parameters were measured in triplicate. On the 7th and 8th February 2024, measurements were recorded within 1 h following the minimum tidal height, at which time the tide pools had been isolated from tidal seawater exchange for several hours. On the 9th February 2024, measurements were taken on the incoming tide (described below). The three tide pools were spatially separated across the rock platform and exhibited no evidence of hydrological connectivity or water exchange between them, and were therefore considered independent spatial replicates.

**FIGURE 1:**
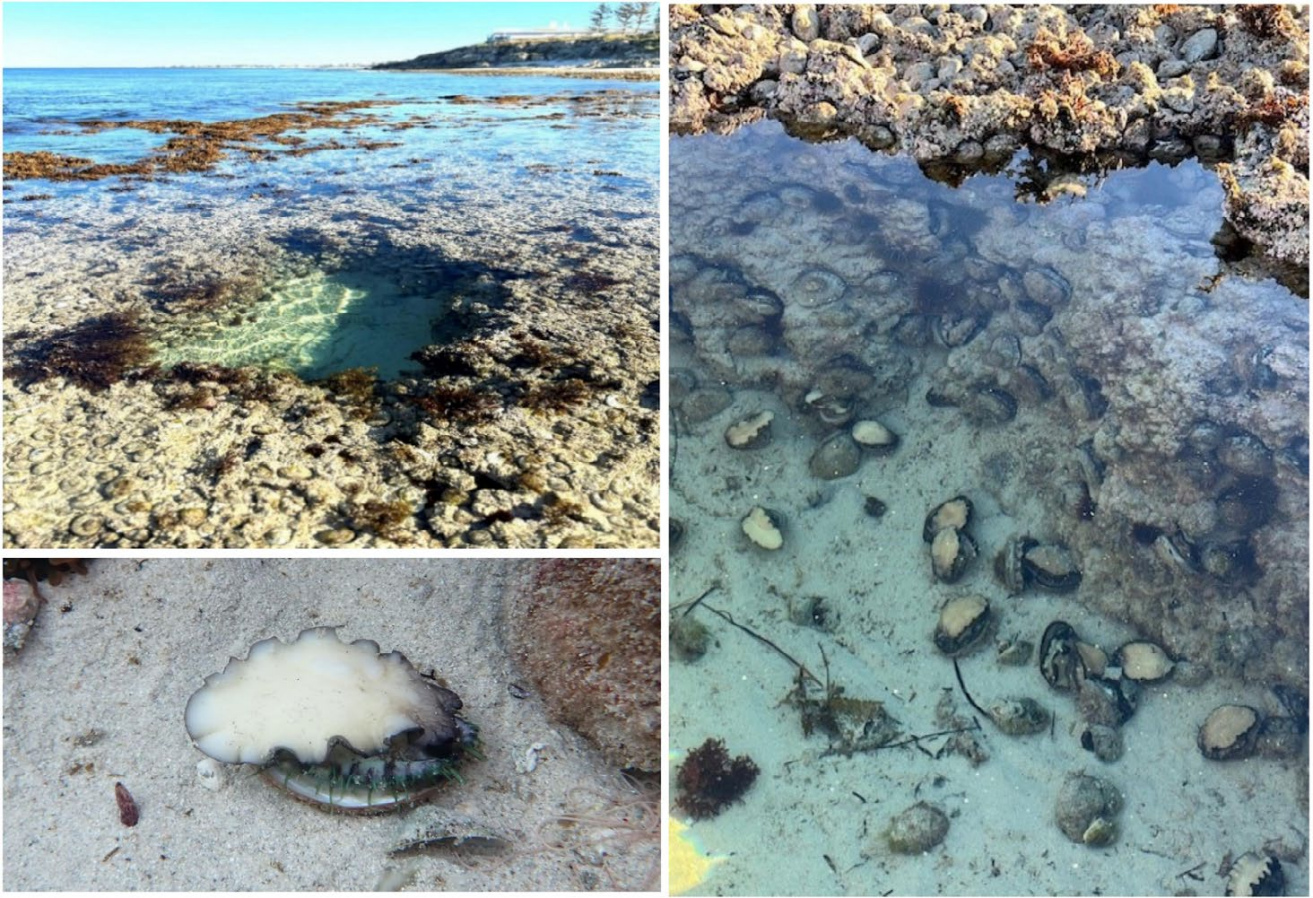
Top left: A small tidepool in the platform at Waterman, Western Australia. Bottom left and right: Moribund abalone in a tidepool at Waterman on 7 February 2024.

On each day, water parameter measurement from the three tidepools was combined and compared to the parameters measured in triplicates at the seaward edge of the platform using a two‐sample Student *t*‐test after verification of normality (Levene's test) and normal distribution (Q‐Q plots). Similarly, the shell lengths of dead or moribund abalone collected from the tidepools were compared with those of abalone observed on the adjacent platform flats using a two‐sample Student's *t*‐test. Statistical significance was reached if *p* < 0.05.

Abalone population size on the Waterman platform was estimated by counting all individuals within ten randomly placed 25 × 25 cm quadrats and extrapolating the resulting numbers to the total platform area inhabited by abalone (720 m^2^).

At the onset of the 2024 heatwave, on 7th February 2024, occasional abalone ( *Haliotis roei* ) were observed on the sand surface in the tidepools, in a moribund condition. On 8 February 2024, all moribund and dead abalone found at the bottom of the tidepools were measured to the nearest 1 mm with digital callipers, as were 162 abalone attached to the rock surface surrounding the tidepools.

Mean water temperatures and salinities at the seaward edge of the Waterman platform were similar to those in the tidepools on all 3 days surveyed in February 2024 (*p* > 0.05 in all cases) (Table 1). On all days there were numerous live abalone attached to the sides of all of the tidepools. On 7 February 2024 the mean dissolved oxygen at the seaward edge of the platform was 74.6% ± 1.1% saturation, but dissolved oxygen was significantly lower (8.3% ± 0.5% O_2_) at the bottom of the tidepools (*p* < 0.001). There were 47 *H. roei* lying on the sand at the bottom of the tidepools (Figure 1). All of the abalone were upside down on the sand. Some were moving but apparently unable to right themselves while others were not moving. All reacted when probed, so they were classified as moribund. On 8 February the oxygen concentration was still significantly lower (7.1% ± 0.6%) in the tidepools than at the seaward margin of the platforms (80.9% ± 0.8%) (*p* < 0.001). There were 7 dead and 31 moribund *H. roei* in the tidepools at Waterman. The *H. roei* were removed from the bottom of the tidepools and measured. Their mean length was 64.4 ± 5.1 mm, not significantly different from the abalone on the platform surface (64.9 ± 2.6 mm) (*n* = 38 and 162, respectively; *p* = 0.814). The moribund abalone from the bottom of the tidepool were placed on the platform surface and were able to reattach firmly. On 9 February 2024, the tide was rising when the initial measurements were made prior to 0645. The mean oxygen concentration in the three tidepools was higher (45.7% ± 10.3%) but was still lower than at the seaward edge of the platform (75.3% ± 0.1%) (*p* = 0.016). A single dead *H. roei* was found at the bottom of the largest tidepool. There were no moribund *H. roei* found in the tidepools on 9th February 2024. By 0700 all three tidepools were flushed by waves on the platform surface and the mean oxygen concentration had increased to 54.2% ± 9.2%.

**TABLE 1:** Mean water temperature, salinity and oxygen saturation at Waterman in February 2024. NM, not measured.

Location	Date	Temp (°C ± SE)	Salinity (±SE)	O_2_ saturation (% ± SE)	Observations
Seaward edge of platform	7 February 24	20.0 ± 0.2	NM	74.6 ± 1.1	1 dead *H. roei* on beach
Tidepools	7 February 24	19.2 ± 0.7	NM	8.3 ± 0.5^a^	47 moribund *H. roei*
Seaward edge of platform	8 February 24	21.3 ± 0.2	37.7 ± 0.2	80.9 ± 0.8	No dead *H. roei* on beach
Tidepools	8 February 24	20.1 ± 0.2	37.0 ± 0.0	7.1 ± 0.6^a^	7 dead *H. roei* ; 31 moribund *H. roei* relocated to open platform at end of survey.
Seaward edge of platform	9 February 24	22.2 ± 0.1	37.3 ± 0.2	75.3 ± 0.1	No dead *H. roei* on beach
Tidepools	9 February 24	21.9 ± 0.2	38.0 ± 0.0	45.7 ± 3.1^a^	No dead or moribund *H. roei*

^a^ Oxygen (% saturation) was significantly lower in tidepools relative to oxygen saturation measured at the edge of the platform (*p* < 0.01 in all cases). Temperatures and salinities were similar in tidepools and seaward edge of platform at all times (*p* > 0.05). *N* = 3 for each seaward edge and *N* = 9 for each tidepool measurement.

The present report is the first record of oxygen deprivation at the bottom of tidepools on the intertidal platforms associated with mortality of abalone and a source of carrion for *Dicathais orbita* (Wells et al. 2026). Elevated water temperature was not directly associated with the observed abalone mortality; however, heatwave conditions may have promoted reduced water exchange and stagnation within tide pools, which, in combination with oxygen depletion, could have acted synergistically to contribute to the mortality event.

The fact that no moribund or dead abalone were observed on the platform surface during the heatwave is interesting as abalone present on the platform were exposed to elevated temperatures and no water exchange for several hours a day during the February 2024 heatwave. It is known that some organisms can survive temperature stress by ‘switching on’ specific genes in response to high temperatures (Gourgou et al. 2010), a phenomenon that has been found ubiquitous in seven species of abalone (Barkan et al. 2025). It is possible that *H. roei* exposed to intense heat stress on the Waterman platform can also upregulate genes to enhance survival chances during heatwaves. Hence, exposed abalone located on the platform surface can survive heat stress with limited water exchange while abalone immersed in the tidepools experience lower ambient temperatures but extreme low dissolved oxygen levels. It is noteworthy to mention that the percent mortality in tidepools represents only a small proportion of the abalone population which is estimated, on this platform, to be *ca*. 96,876 individual abalone. It is therefore unlikely that the dynamics of the local abalone population would be significantly altered by such event.

The oxygen deprivation is a phenomenon worthy of further study. The tidepools are transitory features that occur only in summer when there is a combination of strong easterly winds during the night co‐occurring with low lunar spring tides in the morning. We suggest that respiration by organisms in the pools reduces oxygen levels at the bottom, contributing to abalone mortality, as was observed on 7 and 8 February 2024, although the influence of other co‐occurring environmental stressors cannot be excluded.

## Author Contributions

**Marthe M. Gagnon:** conceptualization (equal), investigation (equal), resources (equal), writing – original draft (equal), writing – review and editing (equal). **Graham L. Cobby:** conceptualization (equal), investigation (equal), resources (equal), writing – original draft (equal), writing – review and editing (equal). **Fred E. Wells:** conceptualization (equal), investigation (equal), resources (equal), writing – original draft (equal), writing – review and editing (equal).

## Funding

The authors have nothing to report.

## Conflicts of Interest

The authors declare no conflicts of interest.

## Data Availability

The data that support this study are included in the paper itself.
